# Case of Destruction of the Kidney by Suppuration

**Published:** 1872-10-01

**Authors:** A. Fisher

**Affiliations:** Chicago, Ill.


### CASE OF DESTRUCTION OF THE KIDNEY BY SUPPURATION.

BY A. FISHER, M.D., CHICAGO, ILL.

On the 13th of April, 1872, I was called to see Mr. P., aged 47 years; temperament nervous and sanguine. I had been his physician for the past 16 years. During that time he had frequent attacks of pain in the left kidney, with difficulty in urinating. About ten or eleven years ago he had acute inflammation of the left kidney, and was very sick for three or four weeks, but finally recovered and seemed in good health, with the exception of pain in the side in the region of the left kidney, with pain and difficulty in voiding his urine, which always caused him more or less trouble. For the last year or two he was up late nights, and drank a good deal of wine and ale, but not to intoxication; his general health was usually good, up to the time I was called to attend him, April 13th. He then said that he was well, but was getting too corpulent, and had his old difficulty of urinating. His respirations were 30 in a minute, short and difficult; pulse 100, and weak; tongue coated and slimy; bowels normal; skin moist, and clammy; urine small in quantity, and loaded with pus (it reddens litmus paper, and he passes it with difficulty. Tests with heat and nitric acid show no signs of albumen); bowels quite full, pressing up the diaphragm, impeding respiration. By manipulating the abdomen, the boundaries of a tumor could be distinctly traced, commencing under the ribs on the left side, about two inches from the median line, and encircling round to the superior spinous process of the left ilium. In every part of this enlargement or tumor, percussion elicited a dull sound, whether lying on his back or either side, whilst there was a resonant sound in every other part of the abdomen. He complained of slight tenderness on pressure over the tumor, more especially near the location of the left kidney. The center of the tumor was rather more yielding or softer than its edges, but it was so dense that fluctuation could not be detected.

Prescribed an emulsion of carb. ammonia and quinine, with diuretics, and painted the tumor with iodine. A similar course was continued until April 21st, with no improvement, the patient gradually growing weaker. At that time, Prof. N. S. Davis saw him with me. He examined the patient carefully, and concluded that he had some kind of a tumor which might eventually suppurate, and thought it a doubtful if not a hopeless case. He recommended a continuation of the treatment, with some slight alterations, which was followed until the 26th, when respiration became very difficult from pressure on the diaphragm, pulse frequent and weak, with subsultus and delirium. I then determined to explore and ascertain whether there was pus in the tumor, and if so, let it out. In the presence of Dr. N. S. Barnes, who assisted me, I introduced an exploring needle an inch and a half deep, about four inches to the left, and a little above the umbilicus, and obtained pus. We then made a longitudinal opening, and let out four quarts of a very fetid pus of a watery character, containing flocculent particles, such as we find in scrofulous abscesses. After the operation the patient was very low and feeble; but by giving tonics and stimulants freely he began to rally, and in 24 hours was much better. Three or four days after the abscess was opened, the same kind of pus passed the bowels with the feces, and at the same time fecal matter was discovered in the pus which came from the abscess, showing a connection between the colon and abscess. Notwithstanding this complication he continued to improve. The fullness of the abdomen subsided; respiration was normal; pulse slower and more full; and he took tonics, wine, and nourishment freely for four weeks or until June the 4th, when irritative fever commenced, with chills; respiration short and labored; pulse frequent and weak; tongue dry and red; with very little discharge of pus either from the opening or bowels. The same difficulty of urinating continued all the time during his sickness. At times during the day his urine appeared normal, and perhaps in two or three hours it would be loaded with pus—proving to my mind that the right kidney was healthy, whilst the left one was destroyed by ulceration; and I gave an opinion to that effect to his wife, two weeks or more before his death, saying that if I was correct, the right kidney would perform the functions of both, and he might possibly recover so far as to be comfortable. From the 4th he sank rapidly, and died on the morning of June 7th.

*Post-mortem* examination was made eight hours after death by Dr. N. S. Barnes and myself. On examining for the left kidney, we found it entirely destroyed by ulceration, nothing remaining of it but the thickened coats, with the descending colon imbedded in them and the surrounding adipose tissue. In the sac or investing membranes we found about a pint of the same kind of pus which we drew from the abscess with traces of feces which passed from the colon through an opening made by ulceration between it and the cavity of the abscess. Adhesions were formed between the sac and peritoneal membrane, for an inch or more around the opening made into the abscess to evacuate the pus. After the pus was drawn off (4 quarts or more), the walls of the abscess, of course, receded, and the adhesions were elongated two inches or more, preventing the pus or feces from entering the peritoneal cavity, still leaving a communication between the abscess and the external opening as well as with the colon and abscess. The ulceration also extended up the crura of the diaphragm into the cavity of the thorax, causing collapse of the left lung by the admission of air from the external opening. We found there nearly a pint of pus of the same character. The lungs were normal, but in the pericardium there were five or six ounces of serum. The right kidney was enlarged, but otherwise appeared perfectly healthy. The coats of the bladder were thickened and contracted. The spleen, being involved in the abscess, could not be found. The peritoneal membrane and intestines were normal, with the exception of that portion of the descending colon that passes over the left kidney. The liver was also normal in appearance and size.

The history of this case shows that suppuration of one kidney may be so chronic and insidious in its progress that the ordinary symptoms which characterize an abscess of that organ may be entirely wanting. I believe that we may date the commencement of the suppuration, at the time or soon after the attack of acute inflammation, ten or eleven years ago; that when the acute inflammation subsided, it left the disease in a chronic form, which gradually terminated in suppuration. For, shortly after and ever since that time, he passed more or less pus from the bladder, and had pain and tenderness in the location of that kidney. It also proves that a person can live with one sound kidney; for after we opened the abscess he improved steadily until it opened into the thoracic cavity, thereby causing collapse of the left lung by the passage of air into the thoracic cavity from the external opening. After that occurrence he sank rapidly, and died in two or three days.

### CASE OF CYSTIC DEGENERATION OF THE KIDNEY.

About twenty years ago I attended a lady in Akron, Ohio, for disease of the left kidney. The case is peculiar, from the fact that two or three times in the twenty-four hours her urine was loaded with albumen, whilst at other times the same day it was normal. After carefully watching the progress of the case for a while, I diagnosed it Bright's disease, or some other organic lesion of the left kidney, with a normal condition of the other, performing the functions of both. At the end of two or three months, no albumen could be detected, and her urine was normal. She finally recovered, and enjoyed good health for four or five years, when she was suddenly taken with acute peritoneal inflammation, and died in four or five days. *Post-mortem* examination revealed the usual appearance of death from peritonitis. There was a pint or more of pus in the peritoneal cavity, with intestinal adhesions, etc. The investing membranes of the left kidney were normal in size and appearance, but were filled with serum, with no vestige of the kidney remaining. The right kidney was much enlarged, but otherwise healthy.

I relate this case to prove that a person can live and enjoy tolerable health for a long time with one healthy kidney; and my knowledge of the fact assisted me in diagnosing the case of Mr. P.

